# Books and Authors

**Published:** 1907-02

**Authors:** 


**Prevalent Diseases of the Eye.** By **Samuel Theobald**, M.D., Clinical Professor of Ophthalmology and Otology, Johns Hopkins University. Octavo of 551 pages, with 219 text-illustrations, and 10 colored plates. Philadelphia and London: W. B. Saunders Company. 1906. (Cloth, $4.50 net; half morocco, $5.50 net).

Among the recent books sent out to the profession this one must be accorded a high place. It is not a book for the specialist, though he may learn much from a study of its pages. It is a treatise for the general practitioner in particular,—a book for anybody who may be called upon to give an opinion concerning a diseased or injured eye, or to treat it.

Theobald has presented a practical treatise on the prevalent diseases of the eye, one that will stand the test of severe scrutiny, one the specialist must approve and the generalist applaud. It may seem odd that a treatise on the eye should appear in these days without a description of the ophthalmoscope and its method of use, yet here is just such a work. The author explains that the great majority of physicians,—general practitioners,—are not skilled in the use of the ophthalmoscope, and are not likely ever to become so, hence it is not worth while to take the time and trouble to deal with such a technical instrument in a book of this character.

It is recommended, instead, that every practitioner likely to be called upon to treat diseased or injured eyes, should acquire the skill necessary to the successful employment of oblique illumination. This is easily mastered and the means are simple, —a reasonably dark room, a light, a biconvex lens two to two and a half inches in diameter, and that is all. In the city a gas jet or an electric lamp can be utilised, in the country a student lamp or even a candle can be employed. Theobald gives instructions as to the method of employing oblique illumination, and illustrates the process by a splendid photographic reproduction. The first two chapters, relating to methods of diagnosis and treatment, abound with common sense and give useful hints relating to each topic.

The chapters that follow deal, respectively, with diseases of the eyelids and orbit, 63-117; diseases of the lachrimal apparatus, 118-150; diseases of the conjunctiva, 151-201; diseases of the cornea and sclera, 202-245; diseases of the iris and ciliary body, 246-274; glaucoma, 275-294; diseases of the crystalline lens and vitreous humor, 295-337; diseases of the choroid coat and optic nerve, 338-387; anomalies of refraction and accommodation, 388-437; muscular anomalies of the eyes, 438-479; injuries of the eye and its appendages, 480-504; and an appendix of approved formulæ, 505-520.

The make-up of the book is such as to please the eye, the columns being slightly narrower than usual, the type larger, and the spaces between the lines wider. We mention these features because they are out of the common order and are excellent. The illustrations add interest to the study of the text. This work takes rank among the foremost books of the year 1906, and will find a lasting place in medical literature.

---

**Diseases of the Stomach.** By **Max Einhorn,** M.D., Professor of Clinical Medicine at the New York Post-Graduate Medical School and Hospital. Fourth revised edition. Octavo, pp. 559. Illustrated. New York: William Wood & Co. 1906. (Price, $3.50).

Ten years and more ago, when Einhorn put out the first edition of this work, it was recognised at once as the most scientific exposition of diseases of the stomach that had been issued in this country. The standard it set up for itself has been maintained through subsequent editions, sent out from time to time as occasion required, the present one being a thorough revision of the whole topic, bringing it down to the year 1906, and including the latest technic of diagnosis and treatment.

It is an interesting fact that the first scientific step in the study of the functions and disorders of the stomach, was taken in this country. Beginning with the famous Beaumont-St. Martin experimental research in 1825, interest was revived in 1867, after a long period of quiescence, when Kussmaul introduced the use of the stomach pump in the treatment of dilatation of the stomach, an instrument made use of later by Leube for the purposes of diagnosis. The names, too, of Ewald, Boas, Reichmann, Riegel and some others are intimately associated with studies relating to functions and diseases of the stomach.

In this country Austin Flint, Delafield, Pepper and others were among the earlier contributors to our knowledge of gastric diseases, and more recently Charles G. Stockton and Allen A. Jones, of Buffalo, Francis P. Kinnicutt, of New York, Hemmeter, of Baltimore, D. D. Stewart, and a number of other investigators have carried the work forward to its present status. But no one, perhaps, has contributed to the brilliant results of the present day in a greater degree than Einhorn himself, and in this treatise he gives the student and practitioner a clear presentation of the subject.

The general appearance of this edition is quite similar to the last one, though several additions have been made. The text has been thoroughly revised and as the work now stands it gives expression to the latest thought concerning the diagnosis and management of diseases of the stomach.

Surgery has contributed not a little in recent years to a better understanding of some gastric conditions, especially ulcer, for which Einhorn gives due credit. Weir, of New York, was one of the first to operate for this lesion. Henry Howitt, of Guelph, Ont., has also operated successfully for ulcer and has contributed importantly to the literature of the subject.

Every one who desires to obtain proficiency in the management of stomach diseases will assuredly derive benefit from a study of the methods set forth by Einhorn in this valuable treatise.

---

**Clinical Diagnosis.** A Textbook of Clinical Microscopy and Clinical Chemistry. By **Charles Phillips Emerson,** A.B., M.D., Resident Physician in John Hopkins Hospital; Associate in Medicine in the John Hopkins University. Octavo, pp. 661. Illustrated. Philadelphia. J. B. Lippincott Co. 1906.

Among the many textbooks relating to clinical diagnosis, some of which are most excellent expositions of the subject, none has dealt with it in quite the same fashion as this one. The author has had a large experience in charge of the clinical laboratory and as instructor in medicine at Johns Hopkins hospital and university, besides having at his disposal the clinical records for seventeen years. The object of the laboratory course at that institution, is not so much to impart knowledge as to raise the efficiency of the student; it is a course not in chemistry and microscopy alone, but in the application of these sciences to the study of the patient himself; not a study in physiology, but one in pathology.

Emerson lays great stress on the examination and study of the sputum, which he says is fast becoming a lost art. This is due, in part, he thinks, to the fact that the discovery of a few specific organisms and the hope of finding more have led to the neglect of the study of the fresh sputum. As a result, the many points which observers of only a generation back were careful to note, are now not looked for at all, or are not seen. The author then proceeds in detail to describe the examination of fresh sputum, for which he says the eyes and nose must be trained as to enable the physician to determine the nature of the case, its stages, or the complication it presents. He appropriates over sixty pages to this description and illustrates them with eighteen figures.

Emerson deals with the urine much in the same fashion, devoting about two hundred and twenty-five pages to the topic. The gastric contents, the intestinal contents and feces, the blood, and, finally, the various body fluids are dealt with in their regular order, the whole making a most instructive and fascinating volume. The chapter on the urine is especially exhaustive and we commend its study to every clinician, as well as to every physician whose duty it is to examine recruits, persons for life insurance, or for any position or occupation where sound kidneys are requisite, or where diseased kidneys must be promptly detected.

The entire work is of unusual value, each section being of special merit. We have simply taken the chapters on the sputum and urine to illustrate the importance of the work in its clinical aspects and to accentuate the author's methods. The book is at once a laboratory working manual and a clinical textbook on diagnosis. It will find its way into every teacher's hands and into every well appointed library.

**Atlas and Textbook of Human Anatomy.** Volume I. By **Professor J. Sobotta,** of Wurtzburg. Edited, with additions, by **J. Playfair McMurrich,** A.M., Ph.D., Professor of Anatomy at the University of Michigan, Ann Arbor. Quarto volume of 258 pages, containing 320 illustrations, mostly in colors. Philadelphia and London: W. B. Saunders, 1906. (Cloth, $6.00 net; half morocco, $7.00 net).

The improvements in the methods of teaching anatomy within the last decade or two have been very marked indeed, to which this atlas and textbook bears abundant testimony. The object lesson afforded by an accurate illustration is one not easily forgotten, and this volume, dealing with the bones, ligaments, joints, and muscles, contains a multitude of beautiful engravings, true to nature and artistic in design and execution. Take the spinal vertebræ, for example, and we do not see how it would be possible to make them clearer, or how even the noviate could fail to understand them as to purpose and mechanism.

The employment of multicolor lithography is made to good purpose; especially does this become a strong feature in the section on myology. The muscles of the trunk are brought out with anatomic and artistic perfection, displaying their origin, insertion, and relations in many instances with more effective instruction than could be done in any other manner. As an example we may refer to figure 236, opposite page 144, which is the most beautiful exhibition of muscle coloring we have seen.

It is unnecessary to pursue the subject further, to indicate the usefulness of the work. The student and practitioner of medicine, for whom it is especially designed, will not be disappointed in the work, which is the most important recent contribution to the literature of human anatomy.

---

**Dose Book and Manual of Prescription Writing:** with a List of the Official Drugs and Preparations, and the more important Newer Remedies. By **E. Q. Thornton,** M.D., Assistant Professor of Materia Medica, Jefferson Medical College, Philadelphia. Third Edition, Revised and Enlarged; Adapted to the New (1905) Pharmacopeia. 12mo, 392 pages, illustrated. Philadelphia and London: W. B. Saunders & Company, 1905. (Bound in flexible leather, $2.00 net.).

A glance at the contents of Dr. Thornton's book fully explains its scope. In addition to the consideration of the official and the more important nonofficial preparations intended for internal administration, weights and measures, solubilities, and incompatabilities, attention is given to the grammatic construction of prescriptions, illustrated by examples. In revising the text for this edition Dr. Thornton has made it to conform with the new (1905) pharmacopeia, the radical change in strength or name of many chemicals, drugs, and preparations already official, and the admission of many newer remedies necessitating the rewriting of a number of sections. We notice in the appendix an addition of much value—a table showing the change in strength of important preparations, and also a list of average doses for adults in accordance with the new pharmacopeia. Dr. Thornton's dose book is, as it always has been, accurate and up to date.

---

**Obstetrics for Nurses.** By **Joseph B. DeLee,** M.D., Professor of Obstetrics in the Northwestern University Medical School, Chicago. Second Revised Edition. 12mo., 510 pages, illustrated. Philadelphia and London: W. B. Saunders Company, 1906. (Cloth, $2.50 net).

In preparing such a work as this,—a treatise on obstetrics for nurses,—it is not easy to determine just how much shall be included, or just how much shall be left behind, pertaining to the science and art of obstetrics. DeLee has been most fortunate in this respect; he has presented enough for the advanced obstetric nurse and has given, as well, the necessary instruction to the beginner in this line of nursing.

Some changes have been made in this edition, notably in adding forty new illustrations and increasing the reading text by the addition of forty-seven pages; in describing the so-called vaginal Cesarean section, and the operation of pubiotomy,—both now quite in vogue, and in discussing the later methods in dealing with obstetric complications, and technic. In its present form it cannot fail to prove useful, also, to medical students and junior practitioners. The illustrations are of a high order, and, altogether, the author has demonstrated his ability as a teacher of the modern obstetric art.

**Practical Dermatology.** A condensed manual of diseases of the skin. By **Bernard Wolff**, M.D., Clinical Professor of Diseases of the Skin in the Atlanta College of Physicians and Surgeons. Large octavo, pp. 288. Illustrated. Chicago: Cleveland Press. 1906.

The author of this book aims to present the salient features of diseases of the skin in such compact form as to meet the requirements of the general practitioner as well as the student of medicine. The several affections are dealt with in alphabetical order. None are treated exhaustively but each is handled with conciseness, the leading features only being given in abstract.

A number of excellent pictures illuminate the text, some being of rare conditions that are seen but once in a lifetime. The shape of the book is awkward, but it is one that contains valuable material which cannot fail to prove helpful to students and junior practitioners, and to all who may wish to consult a manual giving the essentials, when time is too limited to examine an exhaustive treatise. A number of excellent formulæ are given both in the text and in a separate section at the end of the book.

